# Day-to-day progression of vital-sign circadian rhythms in the intensive care unit

**DOI:** 10.1186/s13054-021-03574-w

**Published:** 2021-04-22

**Authors:** Shaun Davidson, Mauricio Villarroel, Mirae Harford, Eoin Finnegan, João Jorge, Duncan Young, Peter Watkinson, Lionel Tarassenko

**Affiliations:** 1 Institute of Biomedical Engineering, Department of Engineering Science, University of Oxford, Oxford, UK; 2 Nuffield Department of Clinical Neurosciences, University of Oxford, Oxford University Hospitals NHS Trust, NIHR Biomedical Research Centre, Oxford, UK

**Keywords:** Circadian rhythms, Intensive care unit, Intensive care, Blood pressure, Vital sign monitoring, Delirium

## Abstract

**Background:**

Disrupted vital-sign circadian rhythms in the intensive care unit (ICU) are associated with complications such as immune system disruption, delirium and increased patient mortality. However, the prevalence and extent of this disruption is not well understood. Tools for its detection are currently limited.

**Methods:**

This paper evaluated and compared vital-sign circadian rhythms in systolic blood pressure, heart rate, respiratory rate and temperature. Comparisons were made between the cohort of patients who recovered from the ICU and those who did not, across three large, publicly available clinical databases. This comparison included a qualitative assessment of rhythm profiles, as well as quantitative metrics such as peak–nadir excursions and correlation to a demographically matched ‘recovered’ profile.

**Results:**

Circadian rhythms were present at the cohort level in all vital signs throughout an ICU stay. Peak–nadir excursions and correlation to a ‘recovered’ profile were typically greater throughout an ICU stay in the cohort of patients who recovered, compared to the cohort of patients who did not.

**Conclusions:**

These results suggest that vital-sign circadian rhythms are typically present at the cohort level throughout an ICU stay and that quantitative assessment of these rhythms may provide information of prognostic use in the ICU.

**Supplementary Information:**

The online version contains supplementary material available at 10.1186/s13054-021-03574-w.

## Background

One of the aims of therapy in the intensive care unit (ICU) is the regulation of vital signs for patients who are acutely unwell, with the systems for maintaining homeostasis often compromised [1]. However, medical interventions to regulate vital signs, combined with patient trauma and a noisy and stressful ICU environment, can severely disrupt a patient’s circadian rhythm [1, 2]. Disrupted circadian rhythms in the ICU are linked to complications such as immune system disruption [3], delirium [4] and increased mortality [5]. Chronically disrupted circadian rhythms are associated with long-term cardiovascular consequences such as stroke and myocardial infarction [6] as well as neurodegenerative diseases such as Parkinson’s disease and dementia [7]. Thus, there is a growing interest in minimising the disruption of circadian rhythms of patients in the ICU [8].

It is difficult to quantify circadian rhythmicity in the ICU [2, 9]. Typical approaches rely upon sleep detection and staging. The gold standard of these methods is polysomnography [2, 9], which requires additional specialised equipment to record and analyse the brain’s electrical activity patterns in patients [10]. Typical sleep staging methods are too cumbersome to be used regularly by clinical staff in the ICU, can further disrupt patients, and are unreliable [9]. Thus, new methods are needed to quantify the level and nature of circadian rhythmicity in the ICU before adjustments to clinical therapy can be made to minimise its disruption [1].

Circadian rhythms, with a 24-h period and varying phase, are present in a variety of vital signs that are commonly measured in the ICU, including systolic blood pressure (SBP), heart rate (HR), respiratory rate (RR) and temperature. SBP and HR typically exhibit elevated levels during the day, with a nocturnal dip and localised peaks in the morning and late afternoon [11, 12]. RR is elevated during the day, with a peak late in the day and a dip overnight [13]. Temperature behaves in a sinusoidal manner, with a trough in the morning and a peak in the late afternoon [12, 14].

Although it is generally understood that patient circadian rhythms are severely disrupted in the ICU, there is limited available quantitative assessment of the degree of this disruption, its daily progression, or the subsequent recovery process. Recent work [12] has shown that, in a cohort of ICU patients who recovered to be discharged home, typical circadian patterns in SBP, HR, RR and temperature were present in the final 24 h of their ICU stay. The presence and progression of these rhythms throughout a patient’s ICU stay, including those patients who died in the ICU, have yet to be established.

We investigated the presence of circadian rhythms in SBP, HR, RR and temperature from the first day of admission to the ICU, comparing the cohort of patients who recovered to be discharged home with those who died or were discharged to hospice care. This investigation was conducted across three large, publicly available clinical databases. We hypothesised that vital-sign circadian rhythms would be largely detectable in both the cohort of patients who recovered from the ICU and the cohort of patients who did not. We further hypothesised that vital-sign circadian rhythms in the cohort of patients who recovered would trend over time towards those of recovered individuals (e.g. increased peak–nadir), while those in the cohort of patients who did not recover would trend away (e.g. reduced peak–nadir).

## Materials and methods

### Databases

This study employed the following three clinical databases:

- Medical Information Mart for Intensive Care III (MIMIC-III), a critical care database gathered between 2001 and 2015 at the Beth Israel Deaconess Medical Centre (BIDMC) in Boston, MA, USA [15, 16].
- The eICU Collaborative Research Database (eICU-CRD), a critical care database gathered between 2014 and 2015 from 208 hospitals across the continental USA [17].
- The Post-Intensive-Care Risk-adjusting Alerting and Monitoring (PICRAM) database (ISRCTN32008295), a critical care database gathered between 2008 and 2015 in the ICU and coronary care unit (CCU) at the John Radcliffe Hospital, Oxford, UK; and between 2009 and 2015 in the ICU at the Royal Berkshire Hospital, Reading, UK.

### Data selection

Data from each database were used to build two cohorts of patients. The first cohort included patients who recovered from the ICU to be discharged home (cohort SRV). The second cohort included patients who either died before hospital discharge or were discharged into hospice care (cohort DCS). These two cohorts were selected following similar criteria to those used by [12]:

1. The patient must have had at least one cuff SBP reading recorded.
2. *Cohort SRV* In the case of MIMIC-III and eICU-CRD, the patient must have been discharged ‘home’ or to ‘home health care’. In the case of PICRAM, the patient must have been discharged with an expected dependency of ‘Able to live without assistance in daily activities’.
  *Cohort DCS* In the case of MIMIC-III and eICU-CRD, the patient must have died or been discharged into hospice care. In the case of PICRAM, the patient must have died or been discharged to palliative care.
3. *Cohort SRV Only* The patient must not have had any do not resuscitate (DNR), do not intubate (DNI), or ‘Comfort Measures Only’ codes, as this indicates a deviation from typical ICU care.
4. The patient must have spent at least 96 h/4 days in the ICU continuously. This criterion was applied as it removed most planned post-surgical ICU stays, resulted in SRV and DCS cohorts of a similar size (see Fig. S1 in Additional file 2) and allowed the comparison of circadian rhythms across several consecutive days with stable patient numbers.
5. Measurements outside of the broad physiological bounds (60 mmHg < SBP < 280 mmHg, 30 bpm < HR < 240 bpm, 4 breaths/min < RR < 60 breaths/min, 34°C < T < 40°C) were excluded.
6. Measurements taken while the patient was under the effect of treatments administered in the ICU that were likely to significantly affect the vital signs being measured were excluded. This process focused on excluding measurements taken while vasopressors, *β*-blockers or other vasoactive medication were active. The exclusion process is discussed in more detail in Davidson et al. [12]. SBP and HR measurements were excluded:
  Up to 1 h after a patient was administered, or the end of infusion of, dobutamine, dopamine, adrenaline/epinephrine, noradrenaline/ norepinephrine, metaraminol, glyceryl trinitrate, dopexamine, nitroprusside, amiodarone, nitroglycerine, nicardipine or isoprenaline [18].
  Up to 2 h after a patient was administered, or the end of infusion of, vasopressin, propofol, magnesium sulphate, ephedrine or phentolamine [19, 20].
  Up to 6 h after a patient was administered, or the end of infusion of, dexmedetomidine or furosemide.
  Up to 24 h after a patient was administered, or the end of infusion of, milrinone, terlipressin, labetalol, metoprolol or hydralazine [18, 21, 22].
7. *Cohort SRV* For MIMIC-III and PICRAM, if the patient had multiple ICU stays within 6 months of each-other, all ICU stays within this period were excluded due to it being unlikely the patient was discharged ‘healthy’. In eICU-CRD, no relative dates were recorded for hospital admissions. Instead, any hospital admission containing multiple ICU admissions was discarded entirely. **Cohort DCS**: Only the final ICU stay prior to death or discharge to hospice care was selected.

### Computing circadian rhythms

For each ICU stay, all measurements in each 1-h period were averaged for each vital sign. This process avoids weighting data towards ICU stays where patients were more ill, and thus likely to have more regular vital-sign measurements. Hourly values were recorded left aligned. (For example, the mean of measurements between 1:00 am and 1:59 am was recorded as occurring at 1:00 am.) Most vital signs were measured at least hourly, with the exception of temperature in MIMIC and PICRAM, which was typically measured once every 4 h. If there were no measurements of a given vital sign in a given 1-h period in an ICU stay, that ICU stay did not contribute any measurement for that hour to the overall analysis.

**Table 1:** Description of component periods in 6-day analysis period from admission

Period	Description
Period I	The 24 h during which patients were being admitted
Period II	The 72 h during which all patients were in the ICU
Period III	The 48 h during which patients began to be discharged or died

**Table 2:** Demographics of the patient cohorts for each database

Cohort	Men^1^				Women^1^			
	No.	Age	LOS	OASIS	No.	Age	LOS	OASIS
MIMIC SRV	1658	56.7 ± 15.7	7.3 ± 4.4	32 ± 8	1048	56.3 ± 16.6	7.5 ± 5.6	32 ± 8
MIMIC DCS	1023	68.5 ± 14.7	12.8 ± 10.8	38 ± 8	862	71.8 ± 14.4	11.9 ± 9.4	39 ± 8
eICU SRV	3175	57.1 ± 15.9	7.4 ± 8.4	30 ± 10	2335	57.6 ± 16.6	6.9 ± 4.0	32 ± 10
eICU DCS	1719	67.3 ± 15.1	10.1 ± 8.1	39 ± 10	1431	68.8 ± 14.7	10.0 ± 11.0	40 ± 10
PICRAM SRV	1082	58.4 ± 16.8	11.6 ± 9.9	38 ± 10	728	57.7 ± 17.1	11.5 ± 10.6^2^	39 ± 10
PICRAM DCS	396	65.3 ± 14.8	13.2 ± 12.2	42 ± 10	215	64.6 ± 15.0	12.0 ± 11.3^2^	44 ± 9

^1^Results presented as mean ± SD.

^2^All metrics apart from LOS in women in PICRAM exhibit significant differences (*p* < 0.01) between the SRV and DCS cohorts for a given database

We decided to analyse vital-sign circadian rhythms for up to 6 days from admission for each patient, as we were interested in the trajectory of these rhythms from admission and the relationship between this trajectory and patient outcome. This decision provided additional days for analysis when patients began to be discharged or died, but ensured at least 40% of each cohort remained throughout (see Fig. S1 in Additional file 2), ensuring that a significant proportion of data remained available for analysis. We divided the 6 days of analysis into three periods, shown in Table 1. These periods are labelled in subsequent figures.

The association between these three periods and the number of available patient measurements can be seen in Fig. S2 in Additional file 2. Period III spans 48 h as a patient with a 4.0 day LOS admitted at 00:00 on the first day will be discharged or die at the beginning of day 4, 48 h before the end of the analysis period. Note that for patients with a LOS greater than 6.0 days, the monitoring period is the first 6.0 days of ICU stay.

Given the established variation in vital signs with age and gender [12, 23], the SRV and DCS cohorts were further broken into both gender and age subgroups. The age subgroups employed were based on those described for ‘health, health services and nutrition—morbidity and handicaps’ in ‘Provisional guidelines on standard international age classifications’ [24] and identical to those in Davidson et al. [12]: 15–44 years, 45–65 years and 65+ years of age.

In addition to basic demographic information such as median age or LOS, we also determined the Oxford Acute Severity of Illness Score (OASIS) [25]. OASIS is a severity of illness score used for predicting patient outcomes and readily determinable using retrospective patient databases as in this study. Common ICU severity of illness scores such as APACHE and SAPS requires a wide variety of information, such as past medical history and comorbidities, which are not necessarily recoverable from retrospective databases, and are thus less suited to this task.

Quantitative assessment of circadian rhythmicity was performed at the cohort level in two ways. First, peak–nadir excursions [26], which provide an indication of circadian rhythm amplitude, were computed for each cohort and each day as:

1 $$\begin{aligned} PN_{VS_{n}} = \mathbf {max}(VS_{n}) - \mathbf {min}(VS_{n}) \end{aligned}$$

where $VS_{n}$ is the set of 24 h vital-sign values for the *n*th day. Second, the Pearson’s correlation coefficient (R) was computed between daily mean 24 h vital-sign profiles within a given cohort. The correlation was computed between each daily vital-sign profile and a demographically matched ‘recovered’ profile. This ‘recovered’ profile was created as follows for a given target cohort:

1. Select patients from the SRV cohort from the same database as the target cohort.
2. Select the subset of these patients from the same gender (Male, Female) and age (15–44 years, 45–65 years, 65+ years) group.
3. Select data from the final 24 h of ICU stay prior to discharge for these patients, as this is the circadian cycle closest to ‘recovery’ for which vital-sign measurements are available.
4. Calculate the ‘recovered’ circadian profile for each vital sign using the selected data.

Correlation coefficients were calculated as:

2 $$\begin{aligned} R_{VS_{n}} = \frac{\mathbf {cov}(VS_{n}, VS_{r})}{\sigma _{VS_{n}} \times \sigma _{VS_{r}}} \end{aligned}$$

where $VS_{n}$ is the set of 24 h vital-sign values for the *n*th day and $VS_{r}$ is the set of 24 hourly demographically matched ‘recovered’ vital-sign values. These quantitative metrics were not calculated for the first day after admission (period I) due to the variable number of measurements available during this period.

## Results

Table 2 summarises the demographic information for the SRV and DCS cohorts in the three databases used. In each case, the mean age of the DCS cohort was at least 6.9 years greater than the SRV cohort. The mean LOS was at least 2.7 days greater, except for the PICRAM database. The mean OASIS scores for the SRV cohort were at least 4 points lower than for the DCS cohorts. Differences in demographics between the SRV and DCS cohorts were smaller for PICRAM than for MIMIC-III and eICU-CRD, and their mean values were greater. A detailed breakdown of the dataset, including the number of patients, hospital admissions, ICU stays and vital-sign measurements that met each data selection criteria is presented in Additional file 1.

**Table 3:** Mean number of vital-sign measurements available per hour over the monitoring period for each database and cohort

Vital Sign	MIMIC-III^1^		eICU-CRD^1^		PICRAM^1^	
	SRV	DCS	SRV	DCS	SRV	DCS
SBP	1287 (31.5%)	811 (28.5%)	4427 (53.2%)	2662 (55.9%)	309 (11.3%)	63 (6.9%)
HR	1898 (46.4%)	1273 (44.7%)	5109 (61.4%)	3067 (64.4%)	1349 (49.4%)	354 (38.4%)
RR	3134 (76.7%)	2304 (80.9%)	5913 (71.1%)	3653 (76.8%)	2101 (76.8%)	777 (84.2%)
Temperature	464 (11.4%)	353 (12.4%)	699 (8.4%)^2^	716 (15.1%)^2^	561 (20.5%)	175 (19.0%)

^1^Results presented as number (percentage of patients with an available measurement).

^2^There is a significant difference ($p < 0.01$) in the mean number of available measurements between the SRV and DCS cohorts for all vital signs and databases apart from temperature in eICU-CRD

Table 3 shows the average number of measurements in each hourly bin and the percentage of patients in each cohort that had an available vital-sign measurement for a given hourly bin. There were fewer SBP measurements available in the PICRAM database compared to the MIMIC-III and eICU-CRD databases, both in absolute numbers and as a percentage. In general, the PICRAM DCS cohort contained a very limited amount of data. Table 3 also shows that the PICRAM DCS cohort, despite its small size, saw a considerably greater decrease in terms of available SBP and HR measurements compared to the PICRAM SRV cohort.

**Fig. 1:**
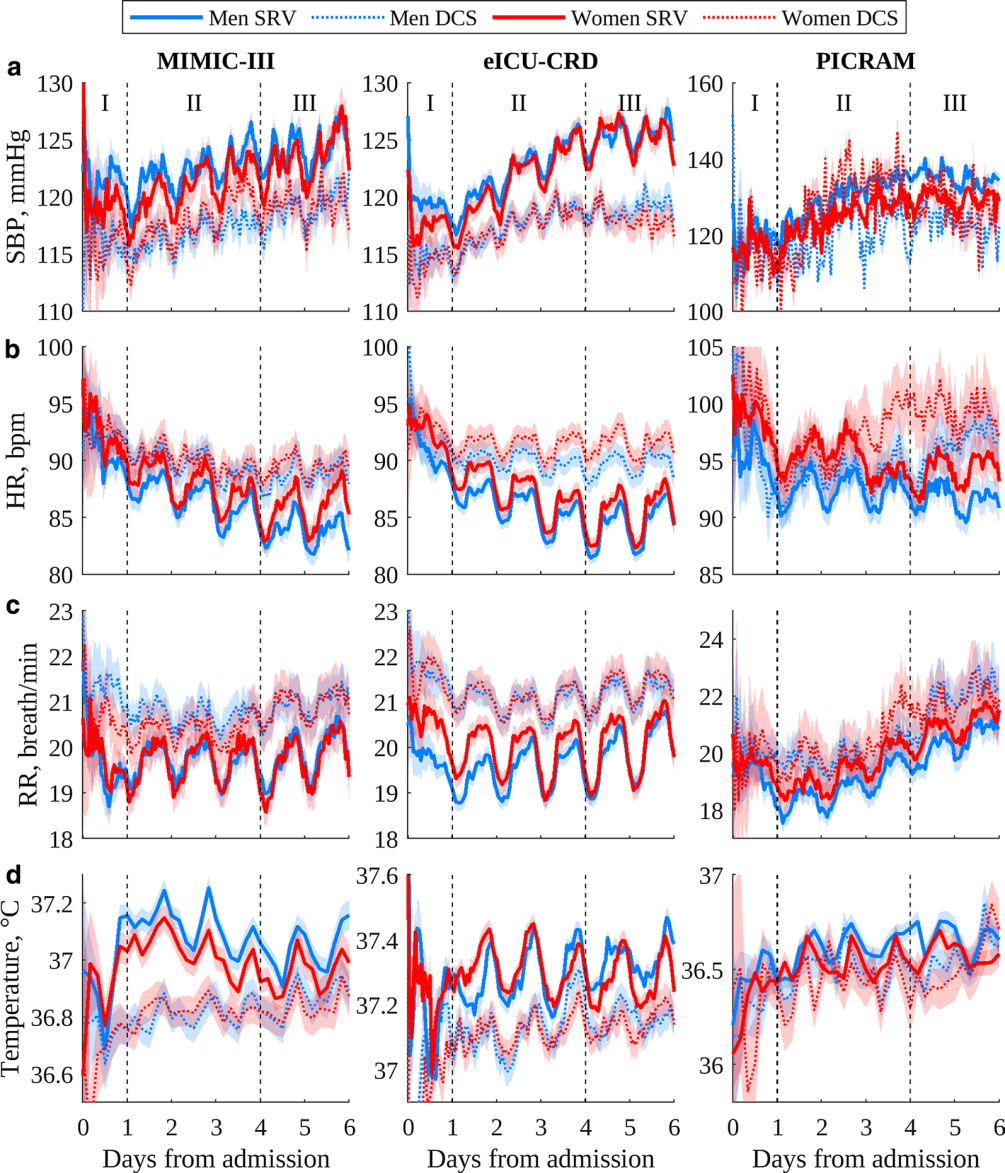
Circadian vital sign plots for the three databases used in the study: **a** SBP; **b** HR; **c**) R; **d** temperature. The 95% confidence interval of the mean is shaded. Note that PICRAM uses different y-axes from MIMIC-III and eICU-CRD due to different mean levels and increased variability

**Fig. 2:**
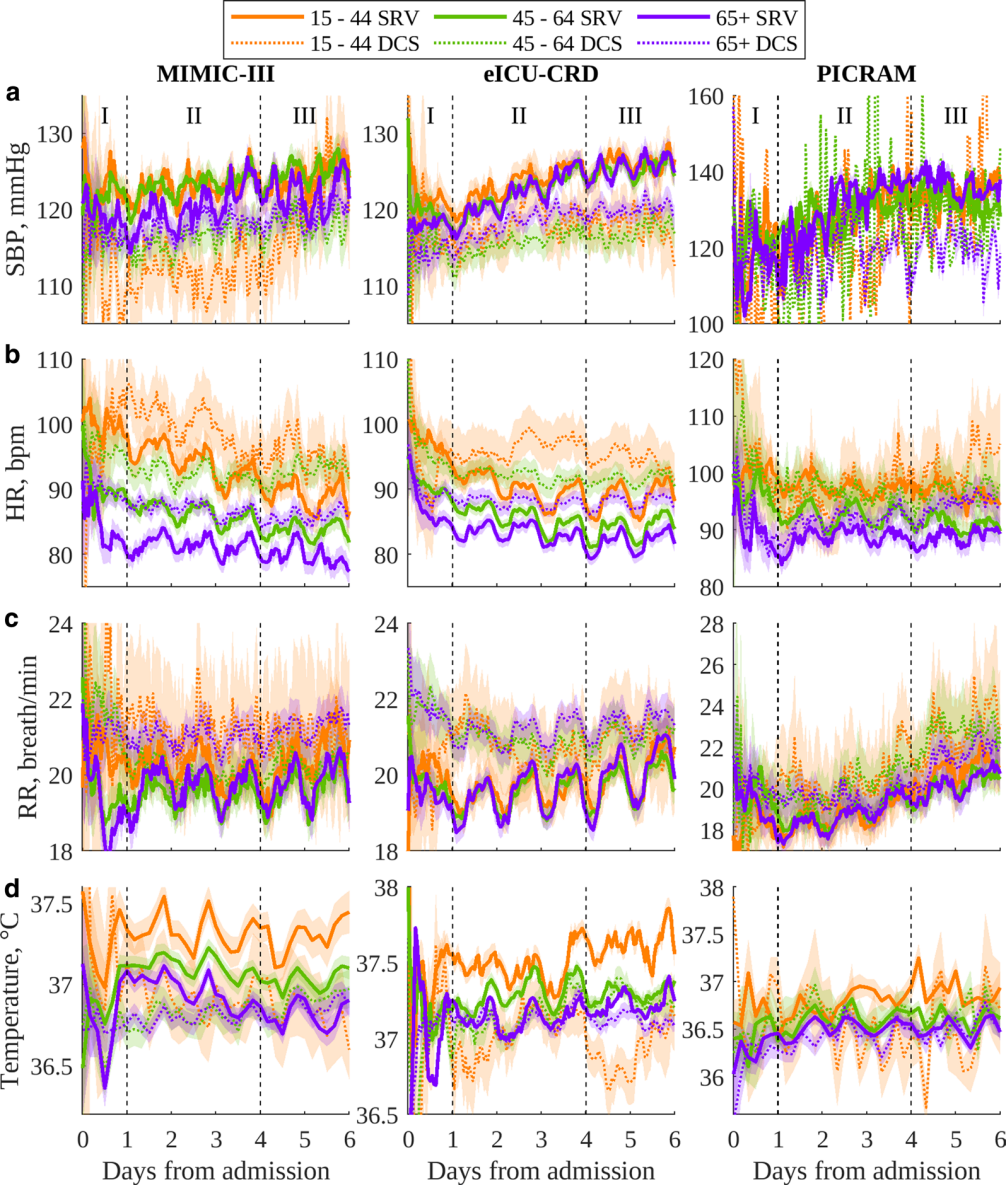
Circadian vital sign plots for ICU admissions of men for each database: **a** SBP; **b** HR; **c** RR; **d** temperature. The 95% confidence interval of the mean is shaded. Note that PICRAM uses different y-axes from MIMIC-III and eICU-CRD due to different mean levels and increased variability

Figure 1 shows the circadian vital-sign profiles in SBP, HR, RR and temperature for men and women in each database. Typical vital-sign patterns in MIMIC-III and eICU-CRD were largely observable in all cohorts throughout the entire period, and in a number of PICRAM cohorts except SBP and the smaller DCS cohorts.

**Fig. 3:**
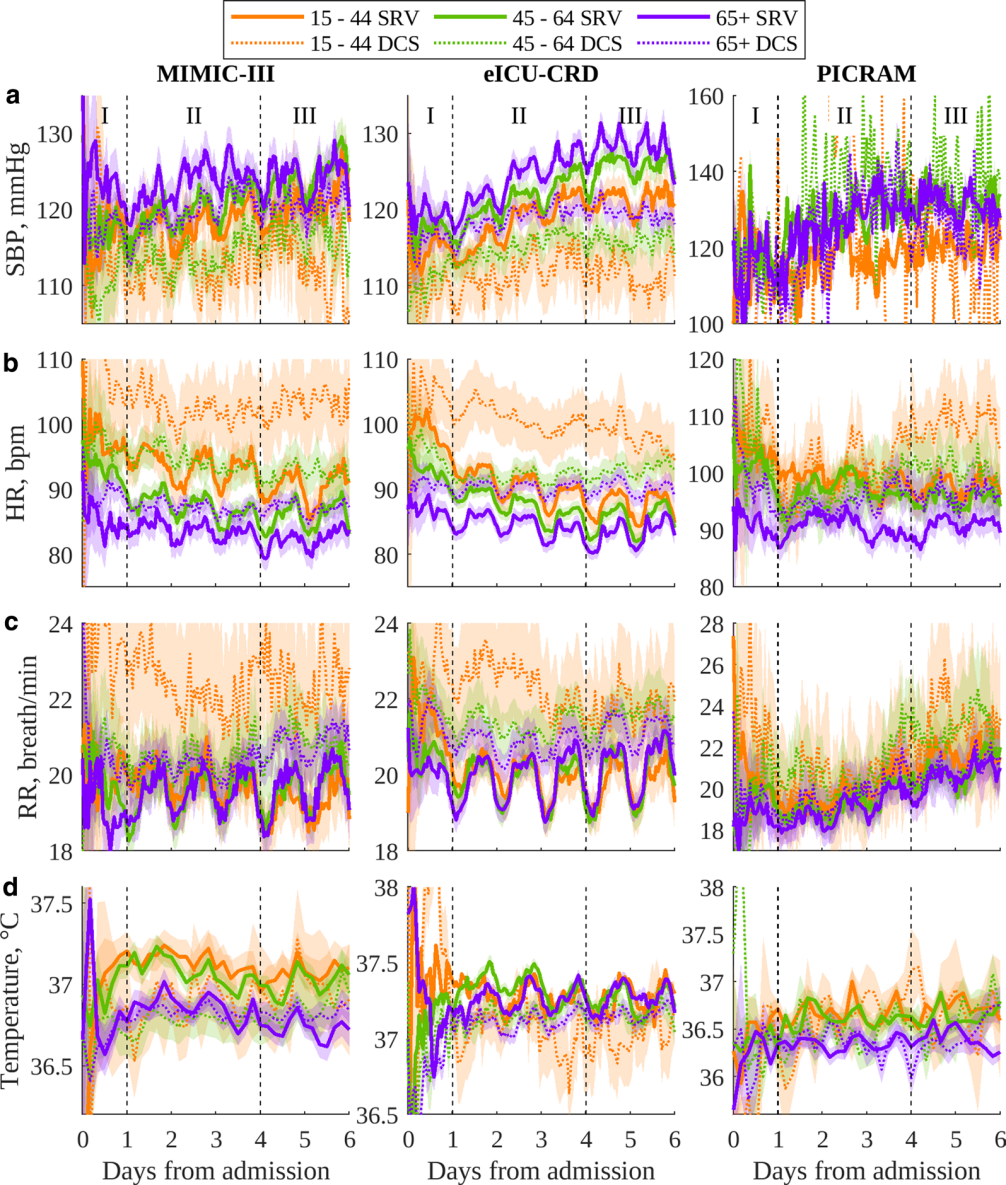
Circadian vital sign plots for ICU admissions of women for each database: **a** SBP; **b** HR; **c** RR; **d** temperature. The 95% confidence interval of the mean is shaded. Note that PICRAM uses different y-axes from MIMIC-III and eICU-CRD due to different mean levels and increased variability

**Fig. 4:**
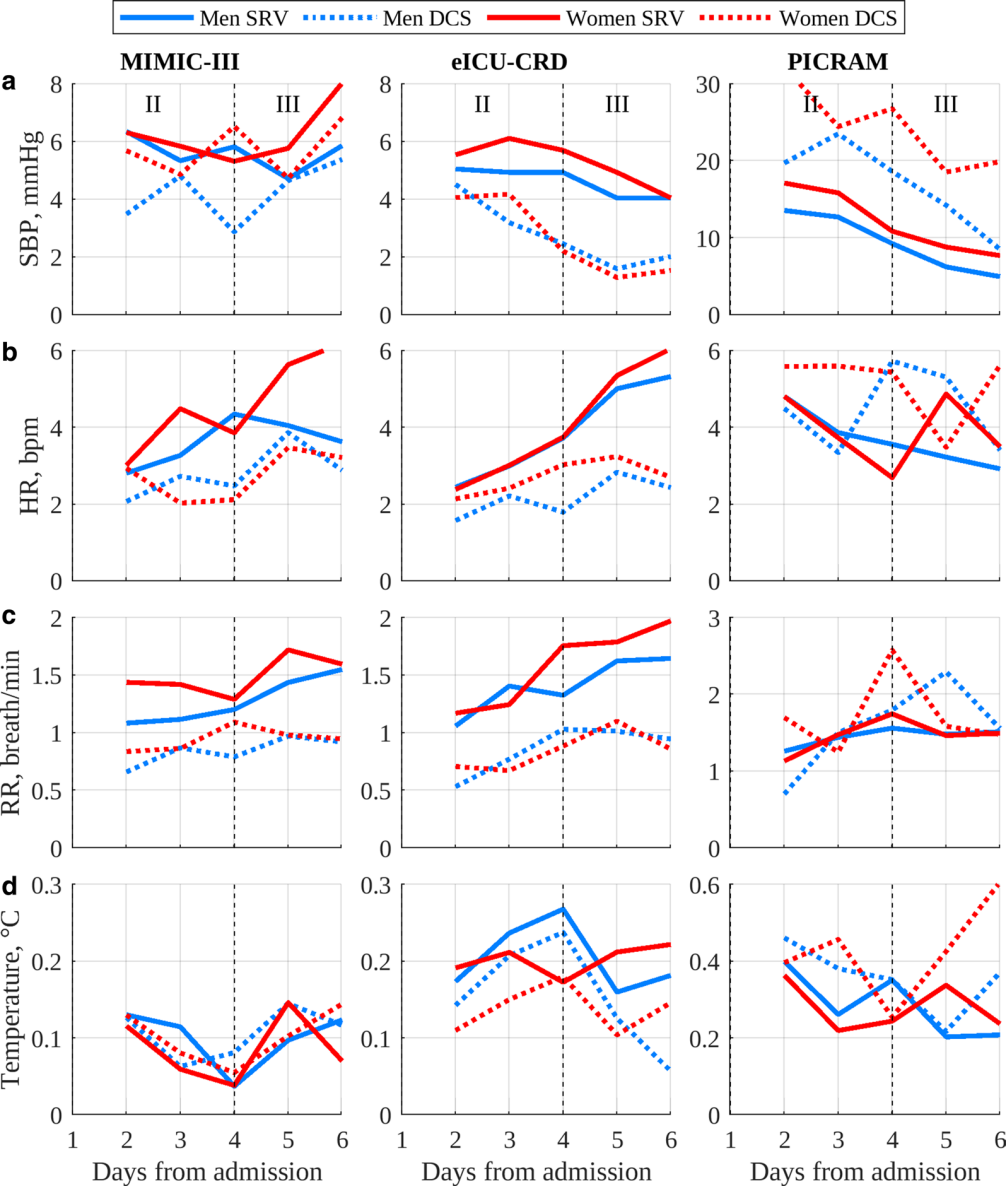
Peak-nadir excursions for ICU admissions in each database: **a** SBP; **b** HR; **c** RR; **d** temperature. Note that PICRAM uses different y-axes from MIMIC-III and eICU-CRD due to different mean levels and increased variability

Figures 2 and 3 show the circadian vital-sign profiles in SBP, HR, RR and temperature for age subgroups in men and women for each database, respectively. In general, typical circadian rhythms, as well as differences in peak–nadirs, means and trajectories were observable in the various age cohorts throughout the entire dataset. Profiles were most clearly observable for the eICU-CRD, but were also observable in the older cohorts for other databases. Results in MIMIC and PICRAM (especially SBP) became increasingly noisy as the cohort size was reduced.

Figure 4 shows the peak–nadir excursions for each vital sign in each database, broken down by gender. In MIMIC and eICU-CRD, the SRV cohort had consistently greater peak–nadir excursions, these peak–nadir excursions typically increased over time. In PICRAM, the DCS cohort typically had greater peak–nadir excursions.

**Fig. 5:**
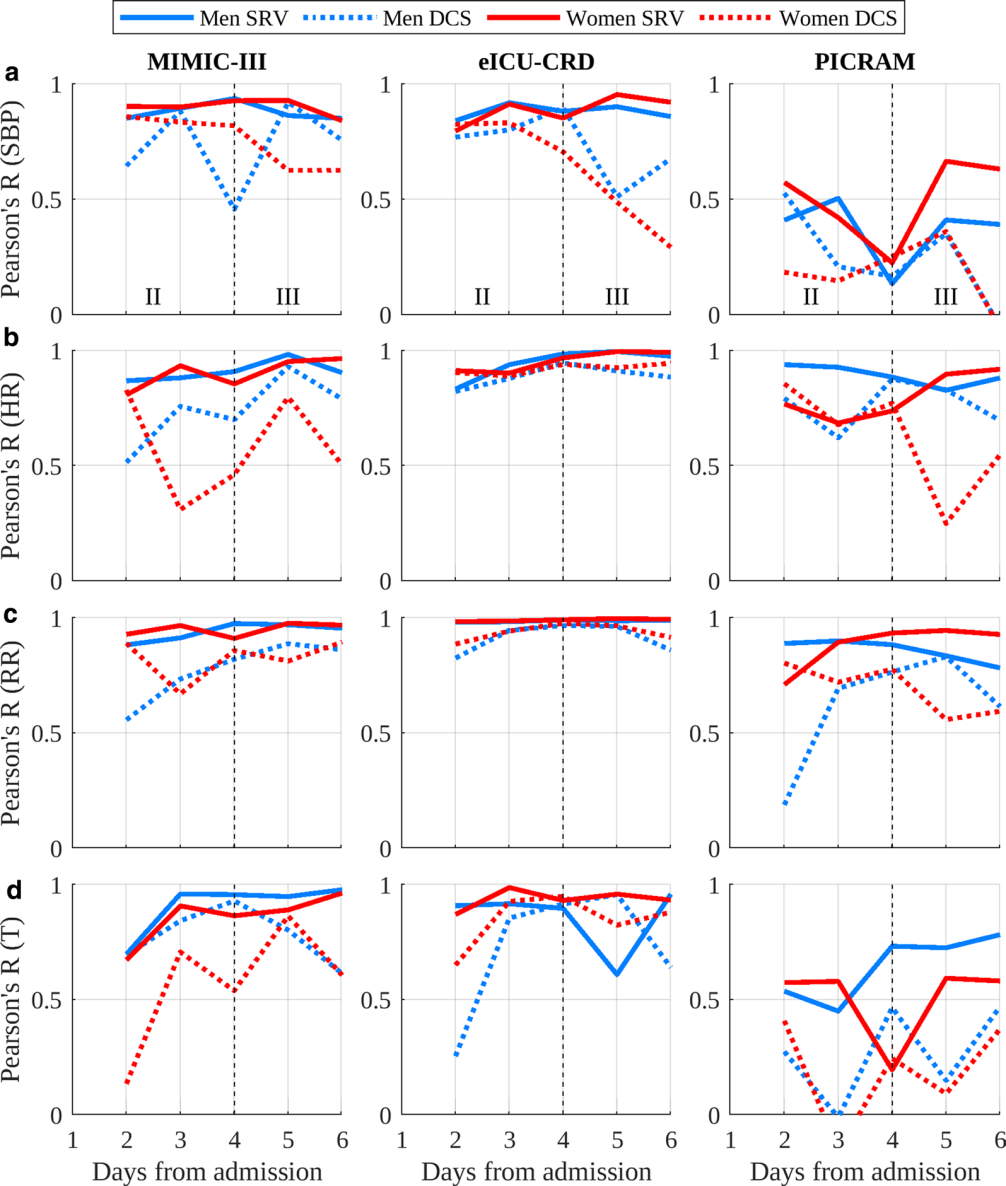
Correlation to healthy final day vital-sign profile grouped by age for each database: **a** SBP; **b** HR; **c** RR; **d** temperature

Figure 5 shows the correlation to a ‘recovered’ final day vital-sign profile for each vital sign in each database, broken down by gender. In all databases, the correlation to a ‘recovered’ final day profile was typically greater for the SRV cohort and typically increased for the SRV cohort over time. For the DCS cohort, correlation tended to be poorer and to decrease beyond day 4.

## Discussion

In this study, we investigated the presence of vital-sign circadian rhythms across multiple days of ICU stay. Data were drawn from three large retrospective clinical databases, and a comparison made between the cohort of patients who recovered and those who did not. Our results suggest that vital-sign circadian rhythms are broadly present at the cohort level throughout an ICU stay and that there is a difference in rhythm profiles between cohorts of patients with differing outcomes.

### Presence of vital-sign circadian rhythms

Figures 1, 2 and 3 show that vital-sign circadian rhythm profiles typical of non-ICU cohorts were present at the cohort level throughout an ICU stay in both patients who recovered and those who did not, though with a suppressed amplitude for the latter group. This result extends the findings of [12], which established the presence of typical vital-sign circadian rhythm profiles in the final day of ICU stay for patients who recovered. In Figs. 1, 2 and 3, consistent elevated SBP, HR, RR and temperature values during the day with peaks and nocturnal troughs were present. Typical vital-sign circadian profiles were not consistently observable in PICRAM, especially for SBP and the smaller DCS cohorts. This lack of observable vital-sign circadian profiles was likely due to the dearth of available data (see Table 3) rather than an underlying difference between the patient cohorts. However, it is worth noting that patients in PICRAM tended to have a greater OASIS score (Table 2) than those in MIMIC-III or eICU-CRD, reflecting the smaller number of ICU beds per capita available in the UK compared to the USA [27, 28]. The number of available measurements on the first day (period I, during which patients were admitted) varied significantly over the day (see Fig. S2 in Additional file 2).

The results shown in this paper are for grouped cohorts of patients, excluding periods of medication that significantly affected the vital signs analysed. 24–34% of patients in the DCS cohort died or were discharged to hospice care in the 4–6-day window (see Fig. S1 in Additional file 2). Thus, the majority of patients remained in the ICU throughout the reporting period. This means that the DCS cohort profiles presented are not representative of individual patients who are heavily medicated and within hours of dying. Such patients would be expected to exhibit significantly altered behaviour, including, potentially, a lack of observable vital-sign circadian rhythms.

There were clinically expected differences in mean levels between the SRV and DCS cohorts for most vital signs. The DCS cohorts consistently showed decreased mean SBP, increased mean HR, increased mean RR and decreased mean temperature, corresponding to the higher sensitivity periods of common early warning scores [29]. These trends were overlaid with expected gender (Fig. 1) and age related (Figs. 2, 3) trends in the mean vital signs, as shown in Davidson et al. [12]. Similar to the results in Pimental et al. [30], mean SBP tended to increase over the course of an ICU stay, and mean HR to decrease, with both of these trends being stronger in the SRV cohort. RR and temperature showed no consistent trends over the course of an ICU stay.

The vital-sign circadian rhythms in our analysis correspond to those reported in the literature for non-ICU cohorts in controlled environments [11, 13, 14], with a suppressed amplitude. They are present across different databases, age and gender cohorts. The three databases included in this study show data gathered over a long period of time at a single centre (MIMIC-III), data gathered over a shorter period of time at a variety of centres (eICU-CRD) and data gathered at a pair of centres in a different country with different standards of care (PICRAM), all of which show broadly comparable circadian patterns and inter-cohort trends. This suggests that the observed patterns are inherent circadian rhythms and not a product of the ICU environment. Cohorts where vital-sign profiles are difficult to interpret are consistently those where there is limited data available, rather than any consistent demographic or treatment-based factors. Thus, these results suggest that vital-sign circadian rhythms are present at a cohort level, throughout an ICU stay.

### Circadian rhythm quantitative metrics

Figure 4 shows that, in general, peak–nadir excursions were greater for the SRV than the DCS cohort in MIMIC-III and eICU-CRD. The peak–nadir excursions in SBP, RR and HR tended to increase over the course of an ICU stay in the SRV cohort, while this behaviour was less prevalent in the DCS cohort. Peak–nadir excursions in vital-sign circadian rhythms have previously been shown to be suppressed in the ICU [12]. Thus, this observed increase in peak–nadir excursions in the SRV cohort over an ICU stay is potentially indicative of a ‘recovery’ of the magnitude of vital-sign circadian rhythms. While the average age of the SRV and DCS cohorts was significantly different (see Table 2), no significant differences in peak-nadir excursions between age groups were noted in Davidson et al. [12], so differing demographics on their own were unlikely to account for the greater peak–nadir excursions in the SRV cohort. Additionally, greater peak–nadir excursions in the SRV cohort compared to the DCS cohort were still present in the age subgroups (see Additional file 4).

It is important to note when interpreting peak–nadir excursions for grouped cohorts of patients that there are distinct known vital-sign patterns in, for example, SBP [6] that are associated with worsened long-term health outcomes. Thus, the lessened peak–nadir excursions in the DCS cohort may represent a cohort with a more diverse range of distinct rhythm shapes or frequencies, as opposed to a homogeneous cohort with lessened peak–nadir excursions. Future work could further cluster patients based on such vital-sign topographies, treating these groups as distinct.

The greater peak–nadir excursions in the SRV cohort compared to the DCS cohort in MIMIC-III and eICU-CRD are not observed for PICRAM. However, the greater peak–nadir excursions observed in the PICRAM DCS cohort, compared to the SRV cohort, are likely due to the relatively small number of measurements available resulting in noisy vital-sign profiles, rather than underlying differences in patient behaviour (see Table 3). This result highlights the weakness of peak–nadir excursions as a metric to quantify circadian rhythm strength in smaller cohorts of patients. Further evidence of the increase in peak–nadir excursions due to noise as cohort size is reduced is provided by the fact that eICU-CRD, the largest database, tended to have the clearest distinction between SRV and DCS cohort peak–nadirs. More evidence still is that the smaller age (15–44) and gender sub-cohorts in each database tended to have less clear distinction in peak–nadirs between the SRV and DCS cohorts (see Additional file 4).

Figure 5 shows that the correlation between 24-h vital-sign profiles and the corresponding recovered final day vital-sign profile provides a potentially more robust metric of circadian rhythmicity than peak–nadir excursions. The SRV cohorts had greater correlation in the majority of cases across all databases, with these correlations increasing over the course of the ICU stay. The DCS cohorts tended to have a lower correlation, and this correlation tended to further decrease after day 4, a trend most consistently visible in eICU-CRD (particularly for HR and RR). These trends were upheld with reasonable consistency across the age subgroups (see Additional file 4). However, the correlation metric penalises noise, as opposed to peak–nadir excursions which ‘reward’ it, thus the smaller size of the DCS cohorts lend themselves to a lower correlation.

Nevertheless, the differences in correlation and peak–nadir excursions between the SRV and DCS cohorts suggest a quantitative difference in circadian rhythms between the cohort of patients who recovered and those who did not. The attenuated amplitude and strength of these rhythms corresponds well to the results found in Davidson et al. [12], where in that study the survivors were the less well cohort and their peak–nadirs were shown to be attenuated relative to those of healthy individuals. This paper additionally shows the relationship between amplitude of circadian rhythms and well-being is monotonic: the strength of the rhythms for those who recovered is greater than for those who died. The combination of the results from the two analyses suggests that tracking vital-sign circadian rhythms throughout an ICU patient’s stay has the potential to provide additional prognostic information over the course of their hospital stay.

### Limitations and future work

There are several limitations of note in this study. All the results presented are for cohorts of patients, rather than individuals. Any individual tracking of patient circadian rhythmicity would require significant additional development of the quantitative metrics presented in this paper.

The analysis presented in this paper necessarily excluded periods where the patient was under the effects of medication that might alter vital signs, such as vasoactive or inotropic medication. The exclusion of such periods was necessary as these treatments could potentially mask underlying vital-sign patterns. Further research would be required to account for the effects of these medications and thus construct patterns of circadian rhythmicity for patients during these periods.

There are demographic differences between the SRV and DCS cohorts. However, this is somewhat minimised by the use of gender and age specific sub-cohorts in the comparisons. Further, the tables in Additional file 3 show that medication and causes of admission are broadly comparable between the two cohorts.

The data employed in this paper are all from large retrospective clinical databases, as opposed to a study with a protocol for evaluating patient circadian rhythms. For example, this study considers the first 4–6 days of ICU stay, which allows us to investigate the initial trajectory of patient circadian rhythms in the ICU. However, patient LOSs varied considerably, with some of the included patients discharged between 4 and 6 days and others remaining in the ICU long after the 6 day mark. Additionally, this data is from two countries (the UK and USA) with somewhat similar demographics. However, this dataset still represents a large cohort spread over two countries and 211 hospitals, with a range of patients, standards of care and clinical practice.

In a retrospective study of a set of databases as large and diverse as these, there are a wide variety of possible approaches for creating and comparing cohorts. In this paper, the decision was made to choose the broadest comparison as a means of establishing the initial prognostic potential of vital-sign circadian rhythms over consecutive days of ICU stay. We compared two non-overlapping cohorts, one exhibiting a robustly coded ‘good’ outcome (the SRV cohort) and the other a robustly coded ‘poor’ outcome (the DCS cohort). Future work could explore a third cohort of patients who were discharged to long-term care facilities or rehabilitation centres. While some information on the association between patient LOS and circadian rhythms can be gleaned from the plots of consecutive days’ rhythms, and the longer average LOS of the DCS cohort compared to the SRV cohort, future work could more directly investigate this association.

Another potential area for future investigation is the complex relationship between sedation, wakefulness, circadian rhythms and delirium [31]. Vital-sign circadian rhythms exist separately from sleep and wakefulness, as shown by these rhythms being observed in studies where healthy volunteers were kept in state of sustained wakefulness with minimal activity [13, 14]. The inherently subjective nature of consciousness scores, combined with intermittent recording and the use of different scoring methodologies between different hospitals across multiple regions, makes it challenging to consistently establish and compare the wakefulness of patients [32]. Some level of standardisation in sleep patterns is inherently imposed by regular shift and meal times in the ICU. Similarly, the complex interaction between sedatives and consciousness makes establishing level of consciousness using medication administered difficult. Thus, categorising or classifying patients in this manner is significantly more difficult than categorising by outcome. Histograms of the most common medications administered to each cohort of patients, including sedatives, are shown in Additional file 3.

An important potential avenue for future work would be to look at the association between specific admission codes, disease states, treatments and circadian rhythms. For example, there are known associations between disrupted circadian rhythms and sedation [31] or conditions such as diabetes [33]. However, there is a significant variety of admission codes and treatments, and there are multiple challenges involved in selecting subgroups, overlap between these subgroups, and data sparsity. For example, the PICRAM database has a limited number of patients with the required instrumentation and length of stay that leads to very noisy vital sign profiles, without any sub-selection based on disease state. Histograms of the most common admission codes for each cohort of patients are shown in Additional file 3.

Ultimately, this study is able to conclude that, on average, patients who died had suppressed circadian rhythms relative to those who did not. The relatively narrow 95% confidence intervals of the means in the vital-sign profiles suggest that these population means are representative and significantly different between cohorts. However, we make no claims as to the cause of this observed effect. Thus, the conclusion of this paper is ‘circadian rhythms are suppressed in those who died relative to those who survived, and thus warrant further investigation as a potential metric of ICU patient’s condition’, but whether this suppression is due to certain aetiologies, ICU treatments, or environmental factors is not established.

## Conclusion

This paper explored the presence of vital-sign circadian rhythms in SBP, HR, RR and temperature across three large retrospective clinical databases. This exploration encompassed multiple days of ICU stay and a comparison between patients who recovered and those who did not. Vital-sign circadian rhythms were found to be present at the cohort level for all groups in all databases. The peak–nadir excursions and correlation to a demographically matched ‘recovered’ vital-sign profile was typically reduced in the cohort of patients who did not recover, compared to the cohort of patients who did. These results suggest that vital-sign circadian rhythms are not as completely disrupted in the ICU as may have been thought and that quantitative assessment of rhythm behaviour may provide additional clinically beneficial information.

## Supplementary information

**Additional file 1**. Measurements and ICU stays that met selection criteria. Description of data: This PDF contains tables of the number of patients, ICU stays, and vital sign measurements that met each SRV and DCS selection criterion in each database.

**Additional file 2**. Number of measurements and ICU stays. Description of data: This PDF contains details of the number of measurements and ICU stays in the selected cohorts available for a given database and vital sign at any given hour.

**Additional file 3**. Diagnoses and medication. Description of data: This PDF contains details of admission diagnoses and medication of the SRV and DCS cohorts of patients from each database.

**Additional file 4**. Quantitative circadian rhythm metrics for age subgroups. Description of data: This PDF contains the results for peak–nadir analysis and correlation to a demographically matched `recovered' circadian vital-sign profile for age and gender subgroups in each database.

## Data Availability

The datasets analysed during the current study are available in the following repositories: MIMIC-III (https://mimic.physionet.org/), eICU-CRD (https://eicu-crd.mit.edu/), PICRAM (http://www.isrctn.com/ISRCTN32008295).

## Abbreviations

ICU: Intensive care unit
SBP: Systolic blood pressure
HR: Heart rate
RR: Respiratory rate
MIMIC-III: Medical Information Mart for Intensive Care III
BIDMC: Beth Israel Deaconess Medical Centre
eICU-CRD: eICU Collaborative Research Database
PICRAM: Post-Intensive-Care Risk-adjusted Alerting and Monitoring
CCU: Coronary care unit
DNR: Do not resuscitate
DNI: Do not intubate
OASIS: Oxford Acute Severity of Illness Score
