# Supplementary material for: Day-to-day progression of vital-sign circadian rhythms in the intensive care unit
Source: Crit Care. 2021 Apr 22;25:156. doi: 10.1186/s13054-021-03574-w (PMC8063456; doi:10.1186/s13054-021-03574-w)
Supplement: Supplementary file 1 — Additional file 1. Measurements and ICU stays that met selection criteria. Description of data: This PDF contains tables of the number of patients, ICU stays, and vital sign measurements that met each SRV and DCS selection criterion in each database. [file 13054_2021_3574_MOESM1_ESM.pdf]

ADDITIONAL FILE 1

Day-to-day progression of vital-sign circadian rhythms in the intensive care unit

Shaun Davidson[1*], Mauricio Villarroel[1], Mirae Harford[1,2], Eoin Finnegan[1], João Jorge[1], Duncan Young[2], Peter Watkinson[2] and Lionel Tarassenko[1]

*Correspondence: shaun.davidson@eng.ox.ac.uk
[1]Institute of Biomedical Engineering, Department of Engineering Science, University of Oxford, Oxford, UK
Full list of author information is available at the end of the article

Measurements and ICU stays that met selection criteria

Tables 1 - 6 show the number of patients (#Pat.), hospital admissions (#Hosp.), ICU stays (#ICU), and vital-sign measurements (#SBP, #HR, #RR, #Temp.) that satisfied the cumulative application of the SRV and DCS selection criteria previously described for each database.

Table 1 The number of patients, hospital admissions, ICU stays, and vital-sign measurements in MIMIC-III that matched each cohort SRV selection criteria.

Subset	#Pat.	#Hosp.	#ICU	#SBP	#HR	#RR	#Temp.
All	46,476	57,786	61,532	2,871,980	7,936,326	6,520,159	1,128,747
1. Cuff SBP	37,671	48,033	51,392	2,871,980	6,168,906	6,433,882	1,114,487
2. Survived	33,483	42,604	45,217	2,455,896	4,991,389	5,158,016	915,228
3. Disch. Home	20,376	24,308	25,169	986,392	1,774,032	1,778,552	322,043
4. No DNR/DNI	20,212	24,039	24,888	970,349	1,736,234	1,740,712	318,767
5. Stay 96h+	3,286	3,487	3,536	333,569	682,601	686,253	130,367
6. Valid Meas.	3,286	3,487	3,536	332,520	682,450	683,303	129,592
7. BP Medication	3,286	3,487	3,536	215,234	364,161	683,303	129,592
8. Day/Night BP	2,721	2,863	2,901	212,111	336,880	571,738	104,986
9. No Repeat Stays	2,639	2,706	2,706	193,078	311,272	530,103	97,402
10. Hourly Avr.	2,639	2,706	2,706	179,712	270,994	433,752	61,872

Table 2 The number of patients, hospital admissions, ICU stays, and vital-sign measurements in MIMIC-III that matched each cohort DCS selection criteria.

Subset	#Pat.	#Hosp.	#ICU	#SBP	#HR	#RR	#Temp.
All	46,476	57,786	61,532	2,871,980	7,936,326	6,520,159	1,128,747
1. Cuff SBP	37,671	48,033	51,392	2,871,980	6,168,906	6,433,882	1,114,487
2. None	37,671	48,033	51,392	2,871,980	6,168,906	6,433,882	1,114,487
3. Died or Hospice	5,965	5,976	6,787	456,837	1,240,489	1,339,866	210,958
4. None	5,965	5,976	6,787	456,837	1,240,489	1,339,866	210,958
5. Stay 96h+	2,769	2,769	2,944	341,240	1,039,203	1,136,356	180,363
6. Valid Meas.	2,769	2,769	2,943	338,334	1,037,625	1,124,765	179,063
7. BP Medication	2,769	2,769	2,943	188,473	453,219	1,124,765	179,063
8. Day/Night BP	1,885	1,885	1,992	184,206	397,580	857,624	125,849
9. No Repeat Stays	1,885	1,885	1,885	171,468	376,759	823,804	119,405
10. Hourly Avr.	1,885	1,885	1,885	157,993	283,971	512,544	77,597

Table 3 The number of patients, hospital admissions, ICU stays, and vital-sign measurements in eICU-CRD that matched each cohort SRV selection criteria.

Subset	#Pat.	#Hosp.	#ICU	#SBP	#HR	#RR	#Temp.
All	139,367	166,355	200,859	22,079,437	146,070,343	128,586,418	13,267,119
1. Cuff SBP	127,486	151,397	176,497	20,666,164	135,849,528	119,354,199	12,444,242
2. Survived	116,598	138,226	160,825	17,370,839	117,960,824	103,466,212	9,673,554
3. Disch. Home	78,489	89,120	101,953	8,119,745	57,392,933	49,471,570	3,637,550
4. No DNR/DNI	74,679	84,478	96,452	7,460,215	53,032,738	45,605,571	3,387,661
5. Stay 96h+	9,310	9,662	9,954	2,569,047	19,348,095	16,576,641	1,762,256
6. Valid Meas.	9,310	9,662	9,954	2,564,469	19,345,816	16,393,269	1,737,881
7. BP Medication	9,310	9,662	9,954	1,416,440	10,115,899	16,393,269	1,737,881
8. Day/Night BP	6,579	6,763	6,941	1,404,184	9,946,620	11,544,226	1,300,971
9. No Repeat Stays	5,378	5,510	5,510	1,109,117	7,786,988	9,084,524	1,071,181
10. Hourly Avr.	5,378	5,510	5,510	577,468	665,471	783,445	91,618

Table 4 The number of patients, hospital admissions, ICU stays, and vital-sign measurements in eICU-CRD that matched each cohort DCS selection criteria.

Subset	#Pat.	#Hosp.	#ICU	#SBP	#HR	#RR	#Temp.
All	139,367	166,355	200,859	22,079,437	146,070,343	128,586,418	13,267,119
1. Cuff SBP	127,486	151,397	176,497	20,666,164	135,849,528	119,354,199	12,444,242
2. None	127,486	151,397	176,497	20,666,164	135,849,528	119,354,199	12,444,242
3. Died or Hospice	13,160	13,171	15,672	3,295,325	17,888,704	15,887,987	2,770,688
4. None	13,160	13,171	15,672	3,295,325	17,888,704	15,887,987	2,770,688
5. Stay 96h+	4,608	4,611	4,930	2,393,594	13,536,442	11,959,833	2,099,621
6. Valid Meas.	4,608	4,611	4,930	2,382,638	13,527,642	11,687,852	2,037,847
7. BP Medication	4,608	4,611	4,930	1,418,442	6,880,215	11,687,837	2,037,832
8. Day/Night BP	3,149	3,150	3,355	1,410,379	6,697,799	8,164,832	1,522,290
9. No Repeat Stays	3,149	3,150	3,150	1,233,678	6,303,657	7,685,515	1,457,804
10. Hourly Avr.	3,149	3,150	3,150	453,234	533,443	658,644	123,818

Table 5 The number of patients, hospital admissions, ICU stays, and vital-sign measurements in PICRAM that matched each cohort SRV selection criteria.

Subset	#Pat.	#Hosp.	#ICU	#SBP	#HR	#RR	#Temp.
All	12,290	13,138	13,949	334,120	1,295,070	1,306,271	346,474
1. Cuff SBP	11,351	12,113	12,845	334,120	1,265,862	1,277,367	341,646
2. Survived	10,034	10,736	11,382	291,376	1,033,072	1,042,400	287,620
3. Disch. Home	7,823	8,249	8,724	195,253	716,926	717,929	200,002
4. No DNR/DNI	7,731	8,149	8,613	188,800	694,441	695,583	195,635
5. Stay 96h+	2,012	2,061	2,154	127,148	494,530	498,006	133,674
6. Valid Meas.	2,012	2,061	2,154	126,676	494,117	497,458	132,926
7. BP Medication	2,012	2,061	2,154	114,694	374,972	497,458	132,926
8. Day/Night BP	2,012	2,061	2,154	114,694	374,972	497,458	132,926
9. No Repeat Stays	1,917	1,937	1,938	101,156	333,322	443,084	117,840
10. Hourly Avr.	1,917	1,937	1,938	99,153	326,866	431,829	116,989

Table 6 The number of patients, hospital admissions, ICU stays, and vital-sign measurements in PICRAM that matched each cohort DCS selection criteria.

Subset	#Pat.	#Hosp.	#ICU	#SBP	#HR	#RR	#Temperature
All	12,290	13,138	13,949	334,120	1,295,070	1,306,271	346,474
1. Cuff SBP	11,351	12,113	12,845	334,120	1,265,862	1,277,367	341,646
2. None	11,351	12,113	12,845	334,120	1,265,862	1,277,367	341,646
3. Died or Hospice	1,566	1,566	1,567	37,207	214,618	216,683	46,990
4. None	1,566	1,566	1,567	37,207	214,618	216,683	46,990
5. Stay 96h+	653	653	653	31,268	182,851	185,968	38,924
6. Valid Meas.	653	653	653	31,065	182,582	185,716	38,329
7. BP Medication	653	653	653	23,905	102,197	185,716	38,329
8. Day/Night BP	653	653	653	23,905	102,197	185,716	38,329
9. No Repeat Stays	653	653	653	23,905	102,197	185,716	38,329
10. Hourly Avr.	653	653	653	23,218	99,387	176,379	37,976

Author details

[1]Institute of Biomedical Engineering, Department of Engineering Science, University of Oxford, Oxford, UK.

[2]Critical Care Research Group, Nuffield Department of Clinical Neurosciences, University of Oxford, Oxford, UK.

References
