# Supplementary material for: Day-to-day progression of vital-sign circadian rhythms in the intensive care unit
Source: Crit Care. 2021 Apr 22;25:156. doi: 10.1186/s13054-021-03574-w (PMC8063456; doi:10.1186/s13054-021-03574-w)
Supplement: Supplementary file 2 — Additional file 2. Number of measurements and ICU stays. Description of data: This PDF contains details of the number of measurements and ICU stays in the selected cohorts available for a given database and vital sign at any given hour. [file 13054_2021_3574_MOESM2_ESM.pdf]

ADDITIONAL FILE 2

Day-to-day progression of vital-sign circadian rhythms in the intensive care unit

Shaun Davidson[1*], Mauricio Villarroel[1], Mirae Harford[1,2], Eoin Finnegan[1], João Jorge[1], Duncan Young[2], Peter Watkinson[2] and Lionel Tarassenko[1]

[*]Correspondence: shaun.davidson@eng.ox.ac.uk
[1]Institute of Biomedical Engineering, Department of Engineering Science, University of Oxford, Oxford, UK
Full list of author information is available at the end of the article

Number of Measurements and ICU stays

Fig. 1 shows the number of ICU stays in the SRV, DCS, and combined cohorts that exceeded a given LOS in days. It is worth noting the number of patients in the SRV and DCS cohorts beyond a LOS of about 4 is more similar, especially for MIMIC-III and eICU-CRD, than the number of patients in each cohort without any restriction on LOS. This effect is significantly less pronounced in PICRAM, where at no given LOS does the DCS cohort become larger than the SRV cohort (i.e. at no LOS is a patient still in ICU more likely to die than recover).

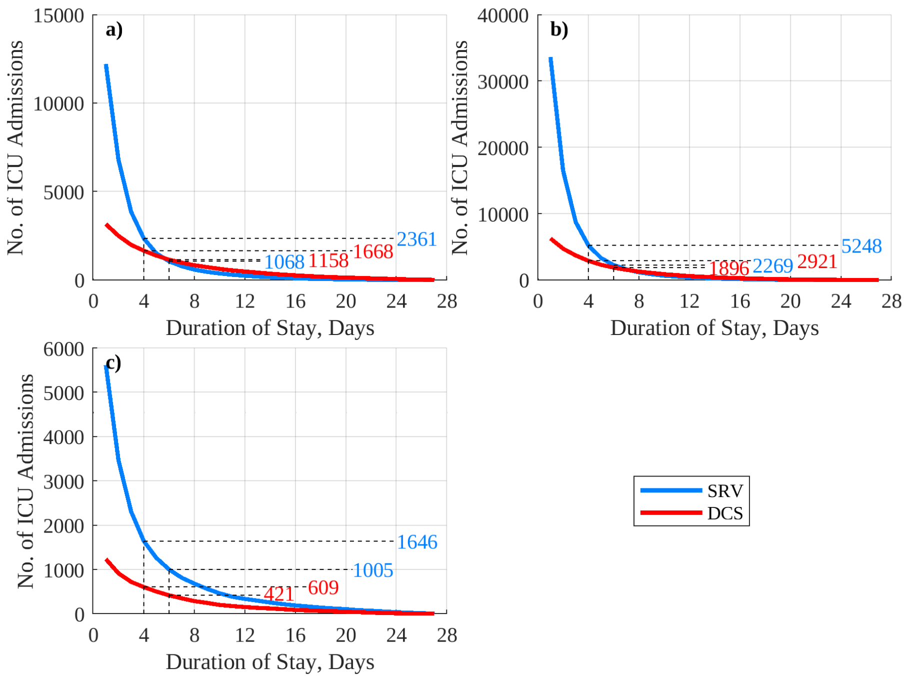

Figure 1 Inverted CDF of patient length of stay in days in each database, broken down by cohort: a) MIMIC-III; b) eICU-CRD; c) PICRAM. The number of patients in each cohort after a minimum of 4 and 6 day LOSs are labelled.

Fig. 2 shows the number of measurements for each vital sign in a given hour for the three databases. The MIMIC-III SRV and DCS cohorts have broadly comparable numbers of vital-sign measurements throughout, while for eICU-CRD there are close to double the number of measurements for SBP, HR, and RR in the SRV cohort. In PICRAM, this difference is further exaggerated with roughly 3 - 5 times the number of measurements in the SRV than the DCS cohort, and significantly fewer measurements overall. The number of SBP measurements in PICRAM is relatively low and concentrated at certain times of day, leading to noisy profiles.

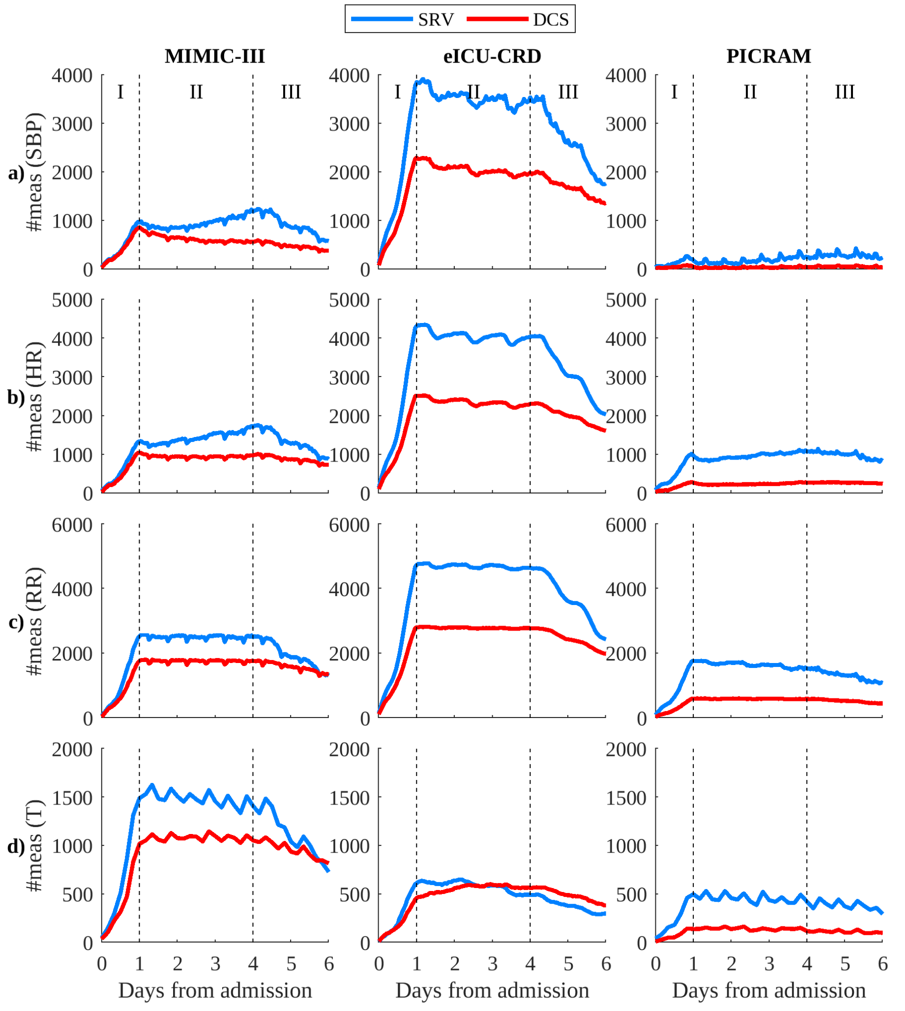

Figure 2 Number of vital-sign measurements for ICU admissions with a LOS of at least 4 days, synchronised to day of admission in each database: a) SBP; b) HR; c) RR; d) Temperature.

Author details

[1]Institute of Biomedical Engineering, Department of Engineering Science, University of Oxford, Oxford, UK.

[2]Critical Care Research Group, Nuffield Department of Clinical Neurosciences, University of Oxford, Oxford, UK.

References
