# Supplementary material for: Day-to-day progression of vital-sign circadian rhythms in the intensive care unit
Source: Crit Care. 2021 Apr 22;25:156. doi: 10.1186/s13054-021-03574-w (PMC8063456; doi:10.1186/s13054-021-03574-w)
Supplement: Supplementary file 3 — Additional file 3. Diagnoses and medication. Description of data: This PDF contains details of admission diagnoses and medication of the SRV and DCS cohorts of patients from each database. [file 13054_2021_3574_MOESM3_ESM.pdf]

ADDITIONAL FILE 3

Day-to-day progression of vital-sign circadian rhythms in the intensive care unit

Shaun Davidson[1*], Mauricio Villarroel[1], Mirae Harford[1,2], Eoin Finnegan[1], João Jorge[1], Duncan Young[2], Peter Watkinson[2] and Lionel Tarassenko[1]

[*]Correspondence:
shaun.davidson@eng.ox.ac.uk
[1]Institute of Biomedical Engineering, Department of Engineering Science, University of Oxford, Oxford, UK
Full list of author information is available at the end of the article

Diagnoses and medication

Figs. 1 - 3 show the 20 most common ICD-9 (MIMIC-III and eICU-CRD) or ICRNARC (PICRAM) codes in the combined SRV and DCS patient cohort. The frequency of occurrence of these codes is represented as the % of patients in each of the SRV and DCS cohorts where a given ICD-9 or ICNARC code was present. Only the top 3 ICD-9 or ICNARC codes per patient were used, to minimise overlapping similar codes being repeatedly counted for a single patient. There are, as one might expect, some notable differences between the databases and between the SRV and DCS cohorts within each databases. However, there is a reasonable degree of overlap in ICD-9 and ICNARC codes between the SRV and DCS cohorts for a given database.

Figs. 4 - 6 show the 20 most common medications administered at least once during an ICU stay in the combined SRV and DCS cohorts in the MIMIC-III, eICU-CRD, and PICRAM databases, respectively. The frequency of occurrence of these medications is represented as the % of patients in each of the SRV and DCS cohorts where a given medication was administered at least once. There are some notable differences in mediation administered between databases, but in general the SRV and DCS cohorts for a given database had similar distributions of administered medication, with more medication administered to the DCS cohort.

Author details
[1]Institute of Biomedical Engineering, Department of Engineering Science, University of Oxford, Oxford, UK.
[2]Critical Care Research Group, Nuffield Department of Clinical Neurosciences, University of Oxford, Oxford, UK.

References

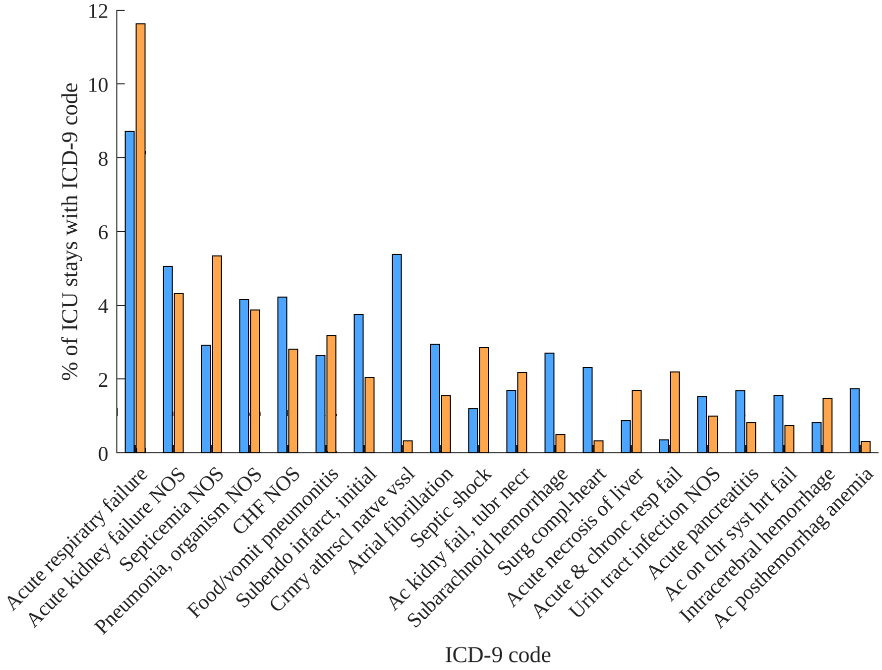

Figure 1 20 most frequent ICD-9 codes for patients in the SRV and DCS cohorts in MIMIC-III

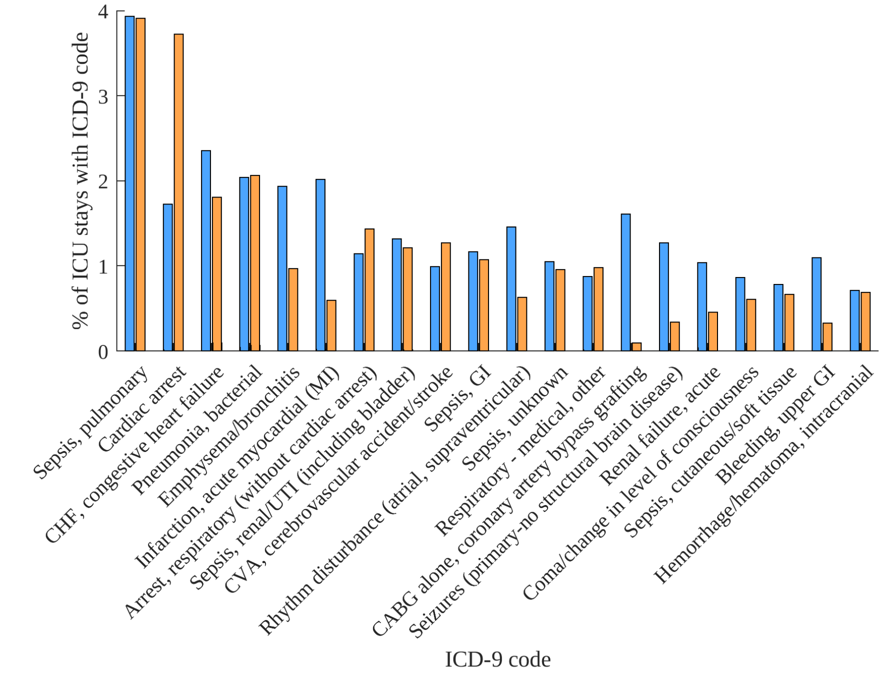

Figure 2 20 most frequent ICD-9 codes for patients in the SRV and DCS cohorts in eICU-CRD

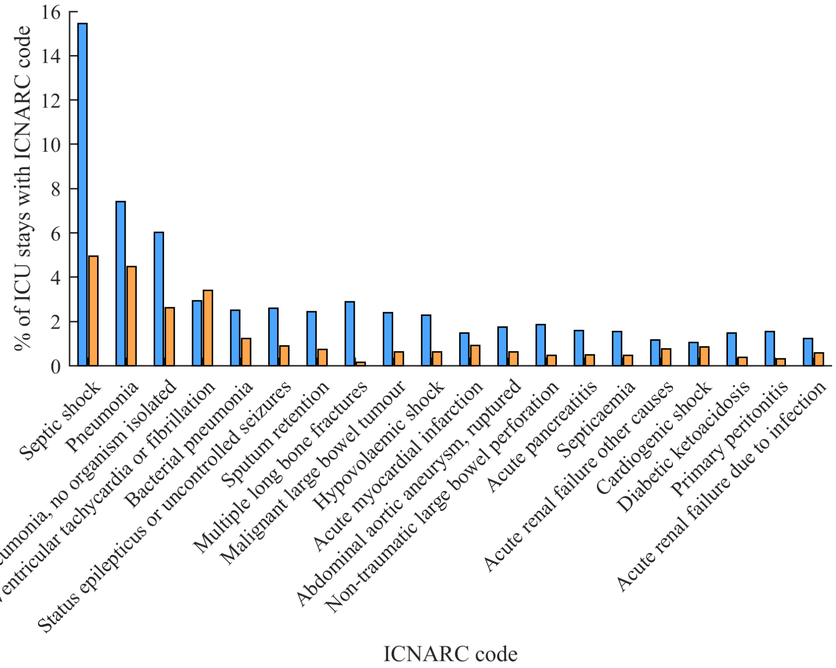

Figure 3 20 most frequent ICNARC codes for patients in the SRV and DCS cohorts in PICRAM

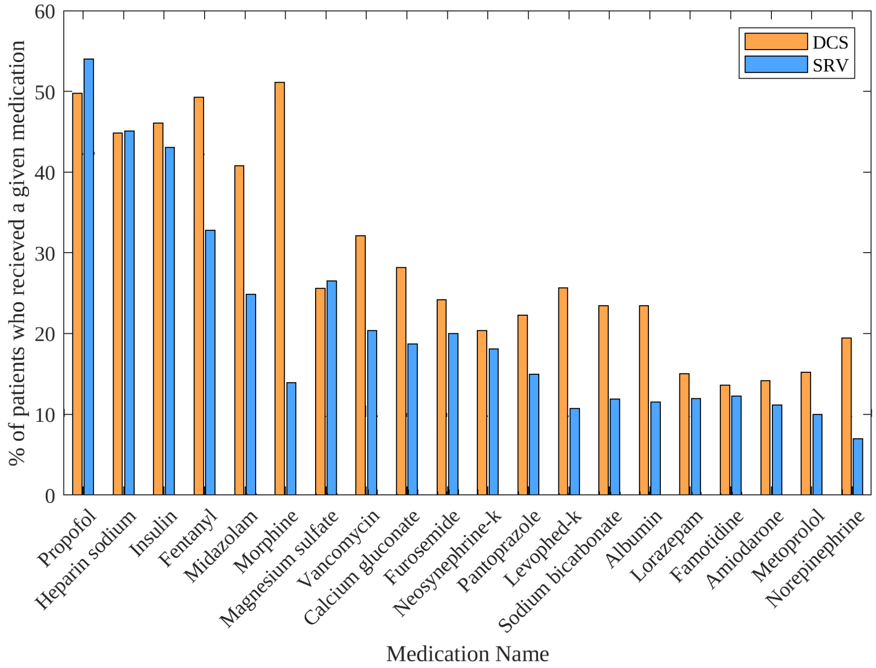

Figure 4 20 most frequent medications administered to patients in the SRV and DCS cohorts in MIMIC-III

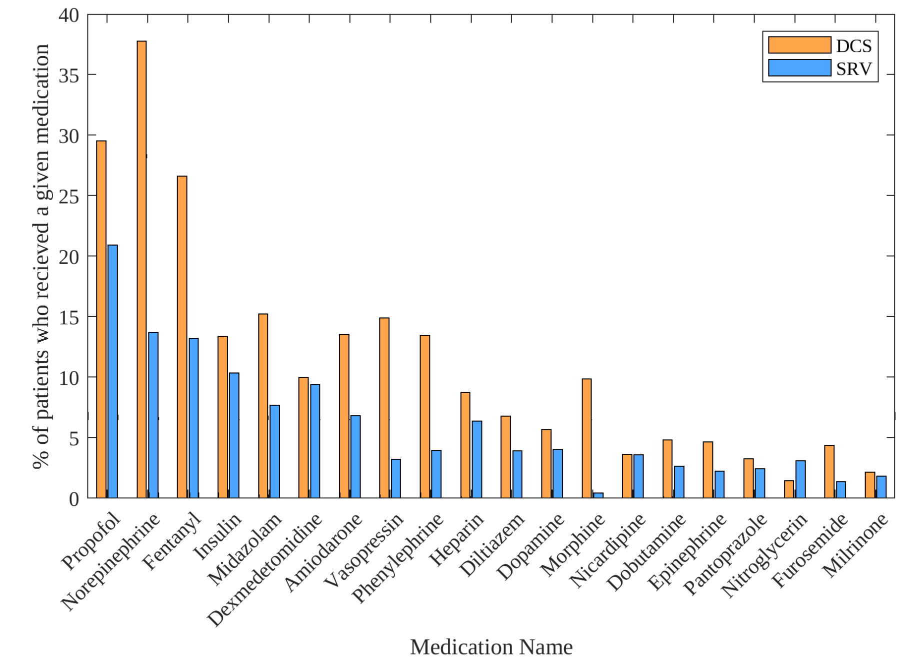

Figure 5 20 most frequent medications administered to patients in the SRV and DCS cohorts in eICU-CRD

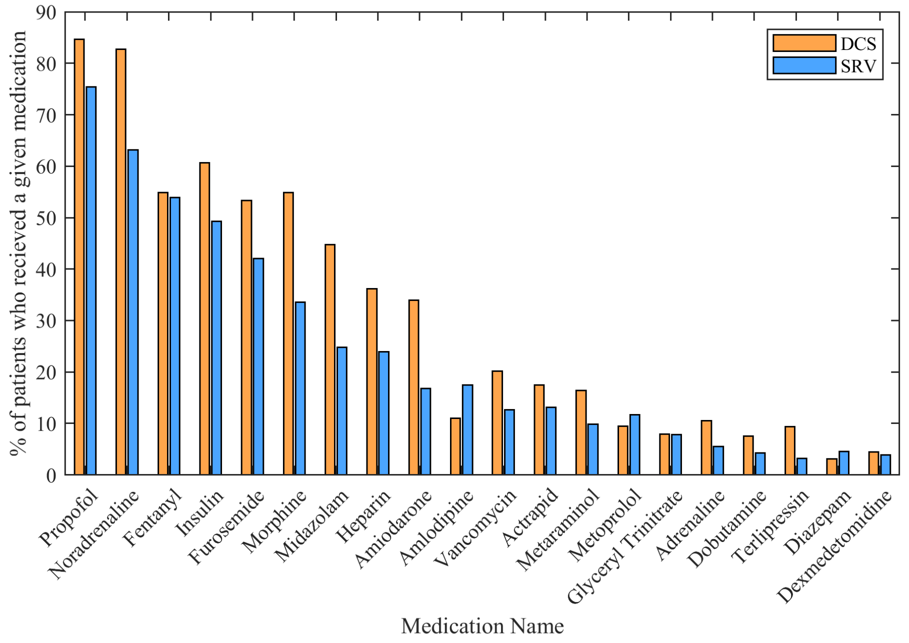

Figure 6 20 most frequent medications administered to patients in the SRV and DCS cohorts in PICRAM
