# Supplementary material for: Day-to-day progression of vital-sign circadian rhythms in the intensive care unit
Source: Crit Care. 2021 Apr 22;25:156. doi: 10.1186/s13054-021-03574-w (PMC8063456; doi:10.1186/s13054-021-03574-w)
Supplement: Supplementary file 4 — Additional file 4. Quantitative circadian rhythm metrics for age subgroups. Description of data: This PDF contains the results for peak–nadir analysis and correlation to a demographically matched `recovered' circadian vital-sign profile for age and gender subgroups in each database. [file 13054_2021_3574_MOESM4_ESM.pdf]

ADDITIONAL FILE 4

Day-to-day progression of vital-sign circadian rhythms in the intensive care unit

Shaun Davidson[1*], Mauricio Villarroel[1], Mirae Harford[1,2], Eoin Finnegan[1], João Jorge[1], Duncan Young[2], Peter Watkinson[2] and Lionel Tarassenko[1]

[*]Correspondence: shaun.davidson@eng.ox.ac.uk
[1]Institute of Biomedical Engineering, Department of Engineering Science, University of Oxford, Oxford, UK
Full list of author information is available at the end of the article

Quantitative circadian rhythm metrics for age subgroups

Figs. 1 and 2 show the peak - nadir excursions for each vital sign in each database, broken down by age and gender. In MIMIC and eICU-CRD, the older SRV cohorts (45 - 65 and 65+) show consistently similar or greater peak - nadir excursions than the corresponding DCS cohorts. However, in the younger 15 - 45 MIMIC and eICU-CRD cohorts, and in PICRAM, the DCS cohort typically has greater peak - nadir excursions due to the reduced cohort size.

Figs. 3 and 4 show the correlation to a 'recovered' final day vital-sign profile for each vital sign in each database, broken down by age and gender. As previously, in all databases, the correlation to a 'recovered' final day profile was typically greater for the SRV cohort, and typically increased for the SRV cohort over time. For the DCS cohort, correlation tended to be poorer, and to decrease beyond day 4.

Author details
[1]Institute of Biomedical Engineering, Department of Engineering Science, University of Oxford, Oxford, UK.
[2]Critical Care Research Group, Nuffield Department of Clinical Neurosciences, University of Oxford, Oxford, UK.

References

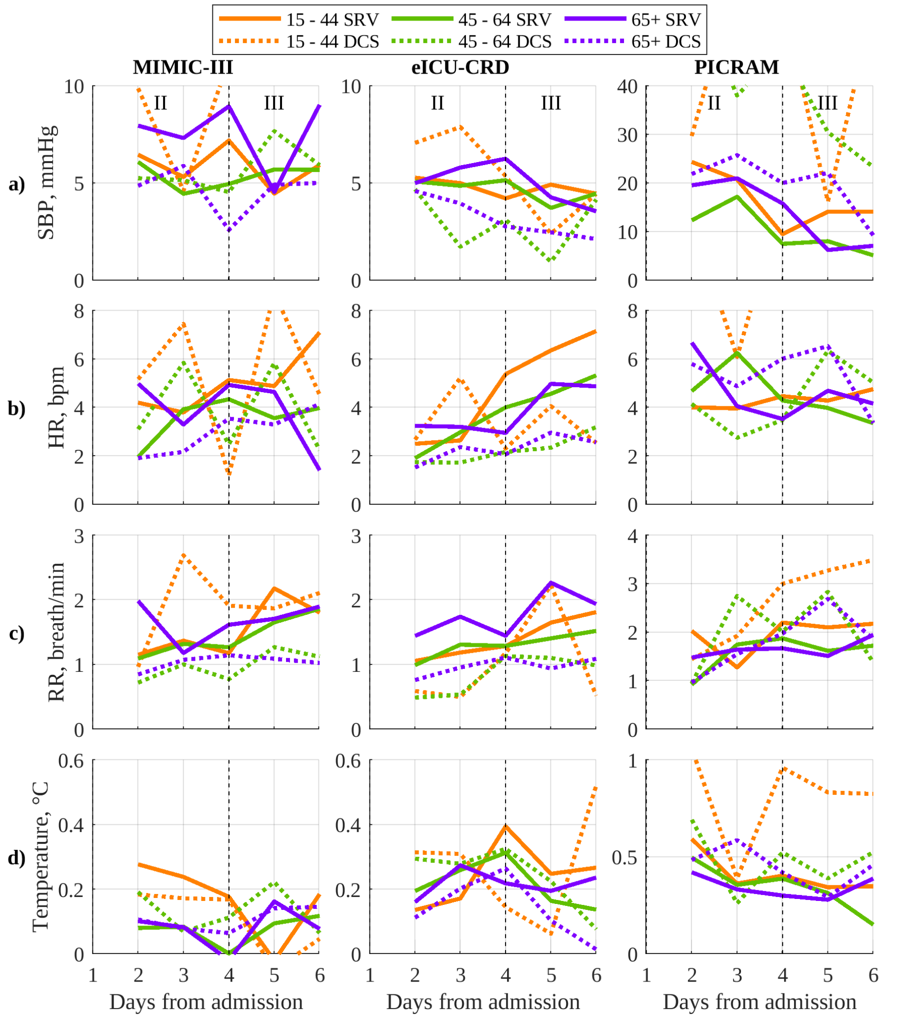

Figure 1 Peak-nadir excursions for ICU admissions of men in each database: a) SBP; b) HR; c) RR; d) Temperature. Note that PICRAM uses different y-axes from MIMIC-III and eICU-CRD due to different mean levels and increased noise.

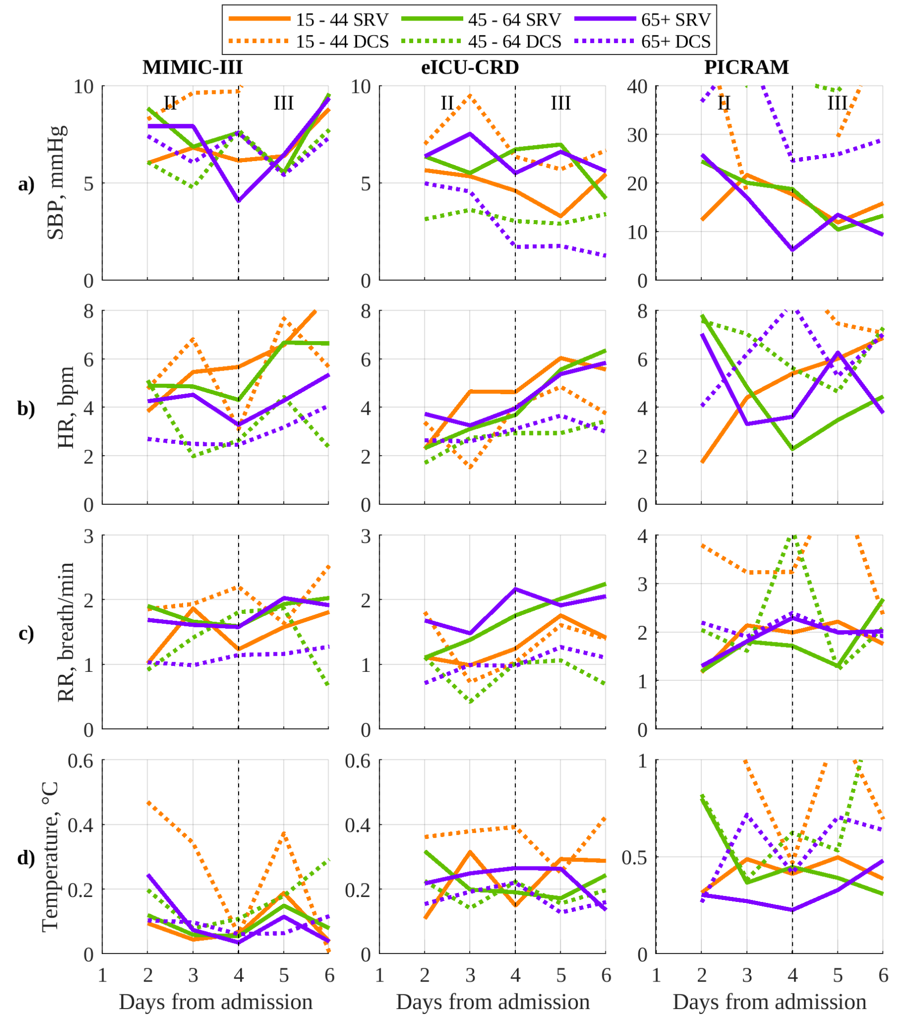

Figure 2 Peak-nadir excursions for ICU admissions of women in each database: a) SBP; b) HR; c) RR; d) Temperature. Note that PICRAM uses different y-axes from MIMIC-III and eICU-CRD due to different mean levels and increased noise.

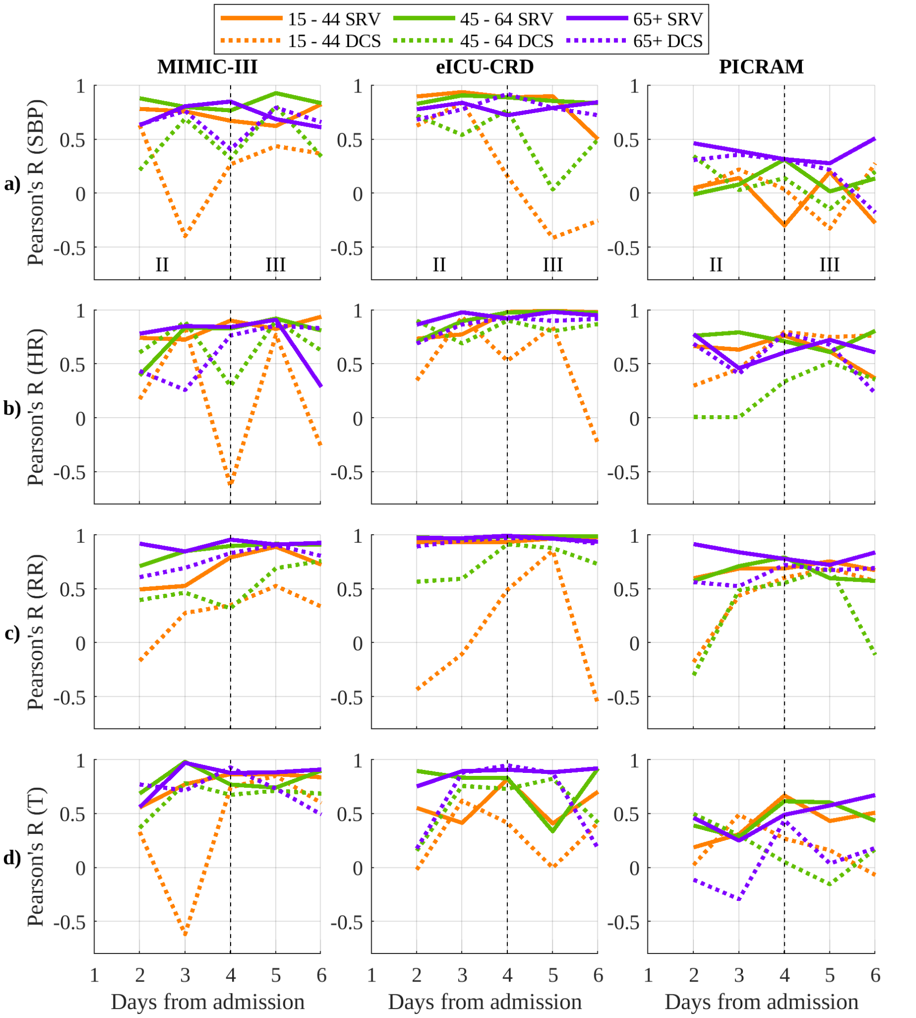

Figure 3 Correlation to recovered final day vital-sign profile for men grouped by age for each database: a) SBP; b) HR; c) RR; d) Temperature.

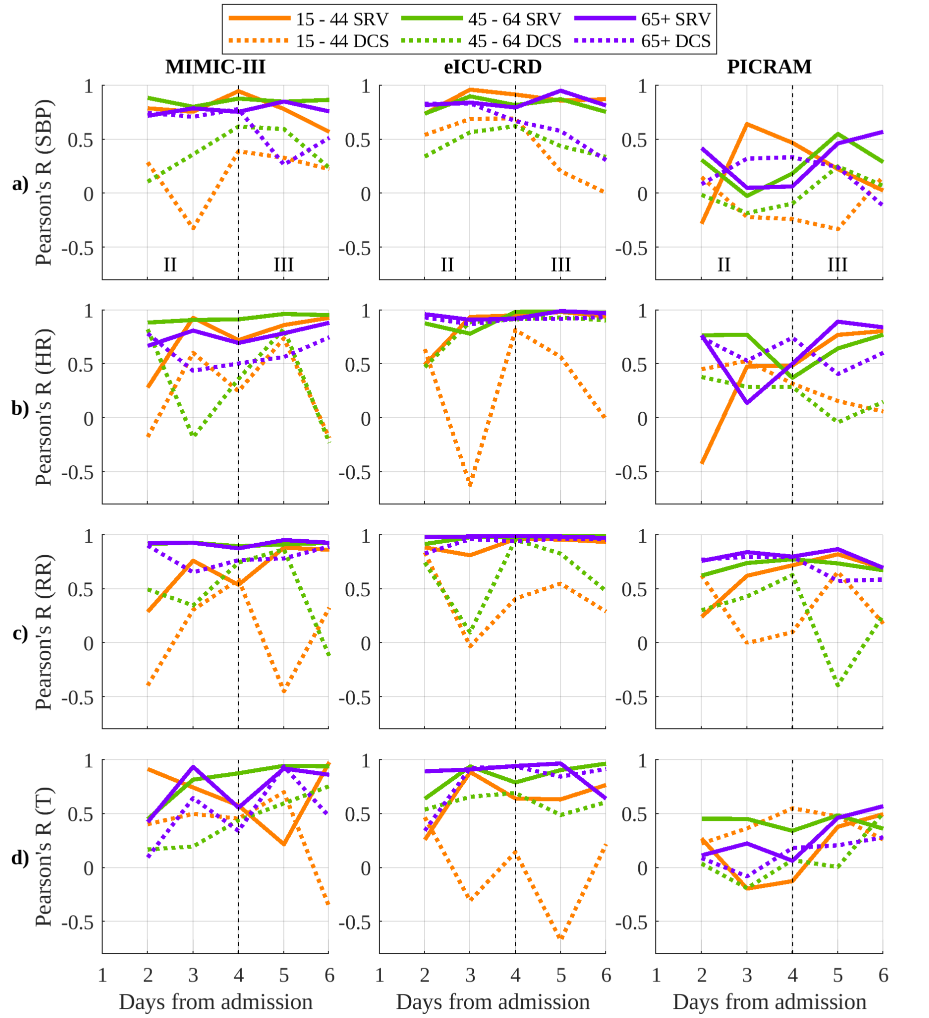

Figure 4 Correlation to recovered final day vital-sign profile for women grouped by age for each database: a) SBP; b) HR; c) RR; d) Temperature.
